# Physicochemical Characterization of *Grewia ferruginea* Hochst. ex A. Rich Mucilage for Potential Use as a Pharmaceutical Excipient

**DOI:** 10.1155/2020/4094350

**Published:** 2020-06-07

**Authors:** Tsadkan Gebremeskel Haile, Gereziher Gebremedhin Sibhat, Fantahun Molla

**Affiliations:** ^1^Department of Pharmaceutics, School of Pharmacy, College of Health Sciences, Mekelle University, Ethiopia; ^2^Department of Pharmacognosy, School of Pharmacy, College of Health Sciences, Mekelle University, Ethiopia

## Abstract

Gum and mucilages from natural sources are in recent times increasingly investigated for pharmaceutical applications. Different studies have shown that the gum and mucilage fraction of various species of the genus *Grewia* were found to be effective viscosity enhancers, stabilizers, disintegrants, suspending agents, gelling agents, bioadhesives, film coating agents, and binders. However, no study has been conducted on the potential use of *Grewia ferruginea* mucilage (GFM) as a pharmaceutical excipient. Therefore, this study was aimed at characterizing the G*rewia ferruginea* bark mucilage for its potential use as a pharmaceutical excipient. The mucilage was extracted from the *Grewia ferruginea* inner stem bark through aqueous extraction, precipitated with 96% ethanol, dried, and powdered. The powdered mucilage was characterized for different physicochemical properties such as powder property, loss on drying, solubility and swelling index, ash value, pH, viscosity, moisture sorption property, microbial load, and acute oral toxicity. According to the results, the percentage yield of the final dried and powdered GFM was found to be 11.96% (*w*/*w*). The density and density-related properties of the mucilage showed good powder flow property. The GFM exhibited pseudoplastic flow behavior. Moisture sorption property of GFM revealed its hygroscopic nature, and its solubility and swelling property was increased with temperature. The pH of GFM was near neutral. Microbial load of the mucilage was within the pharmacopoeial limit, and the oral acute toxicity test revealed that the mucilage is safe up to 2000 mg/kg. From the investigations of this study, it can be concluded that *Grewia ferruginea* bark mucilage has the potential to be utilized as an excipient in pharmaceutical formulations.

## 1. Introduction

Gum and mucilages from natural sources are in recent times increasingly investigated for pharmaceutical applications [1]. This is due to the fact that natural materials are chemically inert, nontoxic, less expensive, biodegradable, and widely available in comparison to the synthetic ones [2].

Gums and mucilages are polysaccharides or complex carbohydrates containing one or more monosaccharides or their derivatives linked with a variety of structures [3]. They can be obtained from different parts of plants, various marine algae, and microorganisms [4]. Those derived from a plant have attracted great interest because of their diverse pharmaceutical applications such as diluents, binders, disintegrants in tablets, thickeners in oral liquids, protective colloids in suspensions, gelling agents in gels, and bases in suppository [5].

**Figure 1:**
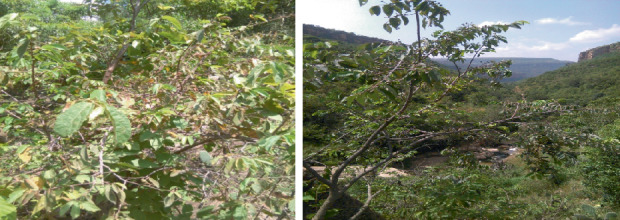
*Grewia ferruginea* Hochst. ex A. Rich plant.

The genus *Grewia* which belongs to the Tiliaceae family is widespread consisting of more than 250 species [6]. It is composed of small trees or shrubs distributed in tropical and subtropical regions of Africa, Arabia, Asia, and Northern Australia [7]. Distribution of the genus *Grewia* in Ethiopia is not extensively studied, but around 11 species, *Grewia ferruginea* Hochst. ex A. Rich, *Grewia schweinfurthii* Burret, *Grewia tenax* (Forssk.) Fiori, *Grewia velutina* (Forssk.) Vahl, *Grewia villosa* Willd., *Grewia bicolor* Juss., and *Grewia mollis* Juss., have been identified and found distributed in different parts of the country [8, 9]. Among these species, *Grewia ferruginea* is one of the indigenous plants in Ethiopia and found in most regions of the country like Tigray, Amhara, Oromia, and SNNPS regions (Figure 1) [10–12].

Mucilage and gum extracts of different *Grewia* species have been investigated as excipients in different pharmaceutical dosage forms [13–16]. Their potential use as viscosity enhancers, stabilizers, disintegrants, suspending agents, gelling agents, bioadhesives, film coating agents, and binders [13, 14, 17–20] has been shown. However, to the best of our knowledge, no report has been established on the potential use of *Grewia ferruginea* mucilage as a pharmaceutical excipient. Hence, the present study was aimed at characterizing the G*rewia ferruginea* bark mucilage for its potential use as a pharmaceutical excipient.

## 2. Materials and Methods

### 2.1. Materials

Hydrochloric acid 35.4% (Loba Chemie, India), sodium hydroxide (Loba Chemie Pvt. Ltd.), ammonium acetate 96% (Loba Chemie, India), methanol 99.8% HPLC grade (Loba Chemie, India), ethanol 96% (Fine Chemicals, Ethiopia), sodium chloride 99.5% (Loba Chemie, India), chloroform (Loba Chemie, India), acetone (Loba Chemie, India), and sulphuric acid (Loba Chemie, India) were purchased from local markets. All chemicals used in this experiment were analytical grade chemicals.

### 2.2. Methods

#### 2.2.1. Sample Collection and Authentication Procedures

The fresh stem barks of *Grewia ferruginea* were randomly collected in the month of December 2017 from the farm lands around Enticho District of the Tigray region in Northern Ethiopia. Plant authentication was made by the Addis Ababa University (AAU) Department of Biology.

#### 2.2.2. Mucilage Extraction and Yield Determination

Extraction of the mucilage was done according to the method described by Ogaji et al. [19]. First, the inner stem bark of *Grewia ferruginea* was peeled off from branches and stem and cut into small pieces with a sharp knife, size reduced, and air-dried for 48 hours. A quantity of 500 g was then added to 15 l of distilled water, soaked for 48 hours, and filtered using a clean muslin cloth to remove debris. 4.5 l of ethanol (96%) was added to the extract to precipitate it, and the precipitate was further treated with another 4.5 l of ethanol (96%) to completely extract the mucilage. Finally, the mucilage was dried in an oven (Beschickug 100–800, Germany) at 50°C for 24 hours, pulverized to a particle size of 180 *μ*m using a miller (ZAIBA OE-999, Japan), and stored in sealed and airtight plastic containers until used for characterization.

The percentage yield of the extracted mucilage was calculated based on the weight of the dried sample used for extraction and the weight of the dried mucilage obtained after extraction as

(1) $$\begin{array}{c} \%\text{mucilage yield}=\frac{\text{weight of dried mucilaginous extract obtained }\left(\text{g}\right)\;}{\text{Weight of dried stem bark powder used }\left(\text{g}\right)}\times 100. \end{array}$$

#### 2.2.3. Physicochemical Characterization of the Mucilage

The dried mucilage was finely powdered in a mortar and pestle, sieved with a mesh size of 180 *μ*m, and characterized for the following properties:

*(1) Bulk Density*. 10 g of the extracted mucilage sample was carefully poured into a 100 ml graduated glass measuring cylinder. The volume occupied by the sample powder was noted, and the bulk density was calculated using

(2) $$\begin{array}{c} \text{Bulk density}=\frac{\text{Average weight of the sample powder}\left(\text{g}\right)}{\text{Volume of the sample powder }\left(\text{mL}\right)}. \end{array}$$

*(2) Tapped Density*. 10 g of powder in a 100 ml measuring cylinder was tapped until the volume of the powder becomes constant. Then, the volume of the powder was recorded, and the tapped density was calculated as

(3) $$\begin{array}{c} \text{Tapped density}=\frac{\text{Weight of the sample powder}\left(\text{g}\right)}{\text{Volume of the sample powder after tapping }\left(\text{mL}\right)}. \end{array}$$

Carr's index and Hausner's ratio were calculated from the bulk and tapped densities according to

(4) $$\begin{array}{c} \text{Car}{\text{r}}^{\prime }\text{s index}\left(\%\right)=\left(\frac{\rho_{\text{T}}-\rho_{\text{B}}}{\rho_{\text{T}}}\right)\times 100, \end{array}$$

(5) $$\begin{array}{c} \text{Hausne}{\text{r}}^{\prime }\text{s ratio}=\left(\frac{\rho_{\text{T}}}{\rho_{\text{B}}}\right), \end{array}$$

where *ρ*_T_ is the tapped density and *ρ*_B_ is the bulk density.

*(3) Angle of Repose*. The angle of repose was measured using the powder flow tester machine (Pharma Test, PT GS4, Germany). Thirty grams of mucilage was placed on the hopper of the machine and allowed to fall under gravity from a nozzle onto a flat surface. Then, the reading for angle of incline of the resultant powder cone was recorded.

*(4) Solubility and Swelling Power*. The method by Takizawa et al. [21] was employed to determine the solubility index and swelling power of the mucilage. Accordingly, 0.125 g sample was dispersed in 10 ml of distilled water contained in a centrifuge test tube. The dispersion was then heated under mild agitation in a thermostatically controlled water bath (WATER BATH, HH-54) at 25°C, 40°C, 55°C, 65°C, 75°C, and 85°C for 10 minutes. The test tubes were then removed from the water bath and immediately immersed in cold water for 5 minutes and centrifuged (CENTRIFUGE, PLC-03, USA) at 3000 rpm for 15 minutes. The supernatant was dried to constant weight in an oven (Beschickug 100–800, Germany) at 105°C. The precipitated paste and the dried supernatant were then weighed. Finally, the water solubility index and swelling power of the mucilage were calculated using

(6) $$\begin{array}{c} \text{Swelling power}=\frac{m_{\text{sw}}}{m_{\text{o}}-m_{\text{s}}}, \end{array}$$

(7) $$\begin{array}{c} \text{Solubility }\left(\%\right)=\frac{m_{\text{s}}}{m_{\text{o}}}\times 100, \end{array}$$

where *m*_sw_ is the weight of the swollen mucilage, *m*_o_ is the sample weight, and *m*_s_ is the weight of the dried supernatant.

*(5) Loss on Drying*. The moisture content of the extracted *Grewia ferruginea* mucilage was determined by the loss-on-drying method as described by Kumar et al. [17]. Accordingly, one gram of the sample was heated at 105°C in a hot air oven (Beschickug 100–800, Germany) until a constant weight was obtained. The percentage loss of moisture on drying was calculated as

(8) $$\begin{array}{c} \text{loss on drying }\left(\%\right)=\frac{\left(\text{initial weight of sample}-\text{weight after drying}\right)\times 100}{\;\left(\text{initial weight of sample}\right)}. \end{array}$$

*(6) Moisture Sorption Properties of the Mucilage*. The moisture sorption pattern was studied at different relative humidity (RH). Pyrex desiccators containing distilled water and saturated solution of NaCl and NaOH at different concentrations (40, 31.58, and 24.66%) were prepared to obtain 100, 75, 60, 40, and 20% relative humidity (RH), respectively, and stored at room temperature. Powder samples were predried in an oven (Beschickug 100–800, Germany) at 120°C for 4 hours. Then, one gram of the predried mucilage was placed on a dried and weighed petri dish and transferred to a particular relative humidity chamber. Samples were equilibrated for 1 week at room temperature. The weights after a week were recorded, and the moisture uptake of each sample was calculated as the average difference of the sample before and after equilibrium in given relative humidity [22].

*(7) Total Ash Value*. 1 g of sample powder was weighed in a preweighed ashing crucible followed by heating in a furnace (CWF12/5, Carbolite, England) at 450°C for 8 hours. Then, the sample was removed and kept in a desiccator and weighed. The total ash value in the sample was calculated using [23]

(9) $$\begin{array}{c} \text{Total ash}\left(\%\right)=\frac{m_{1}-m_{2}}{m}\times 100, \end{array}$$

where *m*_1_ is the mass of the ashing crucible plus the mass of the sample, *m*_2_ is the mass of the crucible plus ash, and *m* is the mass of the sample.

*(8) pH and Conductivity of the Mucilage*. Different masses of mucilage powder (0.1 g, 0.4 g, 0.8 g, and 1.2 g) were dispersed in distilled water at room temperature to prepare 1, 4, 8, and 12% (*w*/*v*) dispersions, respectively. The dispersions were stirred using a magnetic stirrer (DragonLab, MA1F003198, China) for 2 hours. Then, both the pH and conductivity of these dispersions were measured using a calibrated pH meter (AD-8000, Japan) and conductivity meter (professional ATC conductivity meter, Portugal), respectively [24].

*(9) Relative Solubility Test*. The method used by Tadese et al. [25] was employed to determine the relative solubility of the mucilage in cold and hot distilled water, acetone, chloroform, and ethanol. Accordingly, 1 g of the mucilage powder was added to 10 ml of each of the abovementioned solvents and left overnight. Five milliliters of the clear supernatants was taken in small preweighed evaporating dishes and heated to dryness over a thermostatic water bath (WATER BATH, HH-54) at 50°C for organic solvents and in an oven (Beschickug 100 – 800, Germany) at 105°C for distilled water for 2 hours. The weights of the dried residue with reference to the volume of the solutions were determined using an analytical balance and expressed as the percentage solubility of the mucilage in the solvents.

*(10) Viscosity of the Mucilage*. To study the effect of mucilage concentration on viscosity, different concentrations of mucilage powder (2%, 4%, and 6%) were dispersed in 100 ml of distilled water with continuous stirring. The preparations were then kept overnight at room temperature. The viscosities of the dispersions were measured at 20 ± 0.5°C using spindle number 4 of the viscometer (Brookfield RVDVE-8568340, USA) at a shear rate of 20 rpm [26].

To evaluate the effect of the shear rate on viscosity, four grams of mucilage powder was dispersed in 100 ml of distilled water with continuous stirring. Then, the preparation was kept overnight at room temperature. Viscosity of the dispersions was measured at room temperature at different shear rates (20, 50, and 100 rpm) using spindle number 4 of the viscometer (Brookfield RVDVE-8568340, USA) [25].

*(11) Preliminary Phytochemical Screening*. Phytochemical screening of the mucilage for flavonoids, steroidal compounds, alkaloids, and saponins was conducted based on the method described by Tura et al. [27] while tests for tannins were carried out based on the official method in BP [23].

*(12) Acute Oral Toxicity*. The acute oral toxicity study was conducted based on the OECD guideline ([28]. Accordingly, female mice with an average weight of 25 g and age of 6-8 weeks were used in this study. They were housed in appropriate cages, kept at room temperature, allowed free access to water, and fed standard diet until used. The mice were randomly divided into two groups of 5 mice each. Before oral administration, the mice were deprived of food for 3 hours. After three hours, the first group received a single dose of 2000 mg/kg mucilage sample orally using gavage. On the other hand, the second group of mice received only 10 ml/kg of distilled water. Then, the mice were observed for gross behavioral changes for the following 4 hours. The animals were also observed for any symptoms of toxicity and mortality for the following 48 hours. A follow-up for any mortality was continued for 14 days.

*(13) Microbial Loads*. The pour plate method was used to determine the microbial load of the mucilage [23]. Total viable aerobic counts (TVAC) of the *Grewia ferruginea* mucilage for bacteria and Total Combined Mold and Yeast Count (TCMYC) for fungi were conducted using Tryptosan Soya Agar as the medium and Dichloran Rose Bengal Agar medium with 1% Saline-Peptone as diluents, respectively.

For TVAC, one gram of mucilage was suspended in 10 ml of 1% Saline-Peptone solution and serially diluted to get 1 : 10 and 1 : 100. From each of the two dilutions, 1 ml was placed in a sterile petri dish. Then, 20 ml of Tryptosan Soya Agar medium, previously sterilized in an autoclave at 121°C for 15 min, was added and allowed to solidify. For TCMYC, the same procedure was used except the Dichloran Rose Bengal Agar was used as the medium. The Tryptosan Soya Agar medium and combination of 1% Saline-Peptone and Tryptosan Soya Agar medium were used as controls for the bacterial growth, whereas Dichloran Rose Bengal and combination of 1% Saline-Peptone and Dichloran Rose Bengal were used for fungal growth. All the samples were finally incubated in an incubator (UNB oven INB 500, Memmert, Germany) with an inverted petri dish for 48 hours at 37°C for bacteria and for 5 days at 25°C for fungi. Following incubation, the plates were examined for growth and the total colony-forming units (CFU) were counted.

#### 2.2.4. Fourier Transform Infrared (FTIR) Spectroscopy of the Mucilage

The IR spectra of *Grewia ferruginea* mucilage were determined using a Fourier Transform Infrared Spectroscopy (FTIR) spectrometer (SHIMADZU, IR Prestige-21, Japan). A sample of mucilage powder was blended with KBr (potassium bromide) and pressed into pellets under mechanical pressure on the IR press. Then, the films on the disk were scanned over a wave number of 4000-450 cm^−1^ in an IR spectrometer. Finally, the FTIR spectra were recorded and analyzed for the presence of different functional groups [29].

#### 2.2.5. Determination of Total Polysaccharide Content of the Mucilage

A method by Varkhade et al. [30] was employed to estimate the total polysaccharide content of the *Grewia ferruginea* mucilage. First, 10 mg of mucilage was dissolved in 100 ml of distilled water. Then, 1 ml of solution was taken and mixed with 1 ml of 5% phenol followed by the addition of 5 ml concentrated sulphuric acid. Finally, the UV absorbance of the sample was measured using a UV spectrophotometer (T80, PG Instruments Ltd.) against a blank at 488 nm [30].

**Figure 2:**
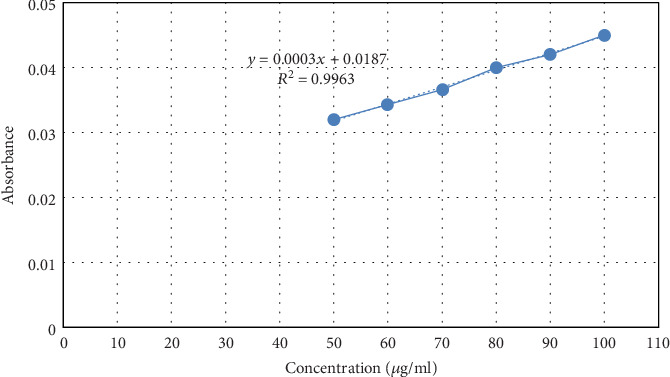
Calibration curve of the standard solution (glucose).

Quantification was made using the equation obtained from the constructed UV calibration curve as shown in Figure 2. To construct the calibration curve, a stock solution of 100 *μ*g/ml glucose in distilled water was prepared initially. Then, aliquots were taken from this solution to obtain sugar concentrations between 50 and 100 *μ*g/ml. 1 ml of 5% phenol solution was added to each sugar solution followed by 5 ml of concentrated sulphuric acid. Then, the absorbance was measured after 10 minutes at 488 nm using a UV spectrophotometer (T80, PG Instruments Ltd.) against a blank.

#### 2.2.6. Statistical Analysis

The results were analyzed statistically using Microsoft Excel and Origin 8 software (OriginLab Corporation). Each test was carried out in triplicate, and the results were reported as mean and standard deviation. At the 95% confidence interval, *p* values less than or equal to 0.05 were considered significant.

## 3. Result and Discussion

### 3.1. Mucilage Yield

The average yield of dried and powdered *Grewia ferruginea* mucilage obtained after extraction was 11.96 ± 1.2%*w*/*w*. The mucilage powder was creamy in colour.

### 3.2. Physicochemical Properties of the Mucilage Powder

#### 3.2.1. Powder Properties

Density and density-related properties are important parameters employed to characterize powder flow properties. Carr's index (%) values from 5-15 and Hausner's ratio values ≤ 1.25 usually account for powders having good flow properties [31, 32]. Angles of repose less than 30° usually indicate a free flowing material, and angles greater than 40° suggest a poorly flowing material [31, 33].

Carr's index (%), Hausner's ratio, and angle of repose of GFM were found to be 13.15 ± 0.1, 1.15 ± 0.03, and 28 ± 0.05 degrees, respectively. Since all these values are within the acceptable ranges, it can be inferred that GFM has a good flow property and could have a potential application in pharmaceutical dosage form formulations. The loss on drying of GFM was found to be 10.85 ± 0.03% which is within the pharmacopoeial specification (maximum of 15%) set for other related excipients like xanthan gum and tragacanth [23]. Lower moisture content in additives is required for safe storage as higher moisture contents can lead to subsequent deterioration in quality and affect the stability of dosage forms containing moisture-sensitive drugs [34]. The total ash value of GFM was found to be 14 ± 0.01%. This value is within the limit of the total ash value specified for xanthan gum in BP which is between 6.5 and 16% [23].

#### 3.2.2. Solubility and Swelling Properties

As depicted in Table 1, the solubility index and swelling power of GFM were increased from 3.2 ± 0.2 to 12.8 ± 0.1 (%)and 64.03 ± 0.01 to 73 ± 0.3 (*g*/*g*), respectively, with increased temperature from 25 to 75°C. The increased swelling power and solubility value with temperature could be due to the weak binding interaction between mucilage molecules at higher temperature, and hence, the mucilage chains enlarge which allows higher water molecule entrapment [29]. Interactions between hydrocolloids and water depend on hydrogen bonding and therefore on temperature [26]. The swelling value in this study revealed that the material under study has good water uptake capacity and swelling property, indicating the potential of the mucilage for use as a release modifier, mucoadhesive, and suspending agent [34, 35]. Consistent results with this finding have been reported on the *Grewia mollis* polysaccharide gum which slowly hydrates and swells in water [36, 37].

#### 3.2.3. Mucilage Moisture Sorption Property

**Figure 3:**
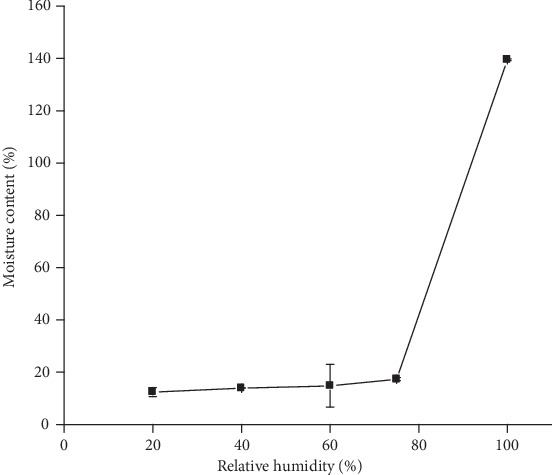
Moisture sorption profiles of the *Grewia ferruginea* mucilage at different relative humidity (mean ± SD, *n* = 3).

Moisture sorption of a pharmaceutical excipient is an important property which has to be characterized since it affects the chemical and physical stability of dosage forms. It has an influence on the packaging and storage of excipients as well as finished products and on the selection of packaging materials [38]. As depicted in Figure 3, the moisture sorption of GFM was increased with increased % RH. Percent moisture sorbed ranged from 12.43 ± 1.76% at low (20%) RH to 139.5 ± 0.37% at high (100%) RH. This implies that the mucilage showed slight increase in sensitivity to moisture in the usual range of relative humidity values but becomes hygroscopic at higher relative humidity values. This result is comparable with the findings reported on moisture sorption properties of cactus mucilage [24].

#### 3.2.4. Relative Solubility Test

Table 2 summarizes the relative solubility of the isolated mucilage in different solvents. The mucilage was found to be insoluble in ethanol, chloroform, and acetone. This reveals that the structural network of the mucilage is composed of hydrophilic chains leading to low affinity for these organic solvents. These results are in agreement with the study done by Ogaji et al. [19] on the *Grewia mollis* mucilage. On the other hand, the solubility profile of the mucilage in water showed first swell-up and then gel formation. This phenomenon might be due to the affinity of the polysaccharides in the mucilage for water through their hydrophilic groups.

#### 3.2.5. pH and Conductivity of the Mucilage

The effect of mucilage concentration on pH and conductivity is presented in Table 3. Accordingly, increased mucilage concentration from 1 to 12% increased the conductivity from 1.94 to 22.2 while pH values slightly decreased. At all concentrations of the mucilage, the pH was found to be near to neutral pH which implies that the mucilage would be less irritating to the mucous membrane of the GIT and it can be suitably employed for formulations which are preferably stable at this pH. The increased conductivity with increased concentration of the mucilage might be due to the presence of mono- and divalent electrolytes in the mucilage. Similar findings have been reported on the conductivity of cactus mucilage [24].

#### 3.2.6. Viscosity of the Mucilage

**Figure 4:**
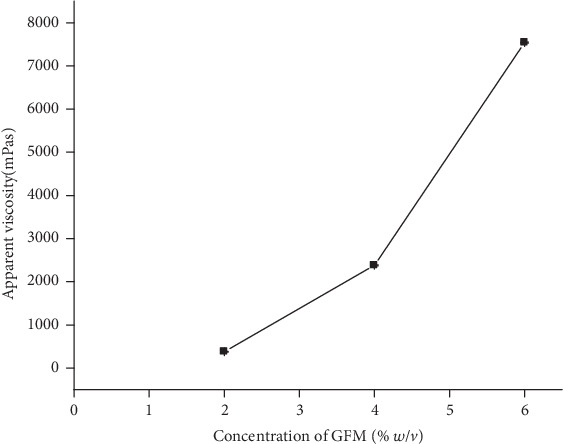
Effect of mucilage concentration on apparent viscosity of its dispersions at 20 rpm shear rate.

*(1) Effect of Concentration on Viscosity*. Viscosity is a desired quality parameter of excipients intended to be used in liquid dosage forms. As shown in Figure 4, the viscosity of the mucilage was increased from 380 to 7540 mPas with increased mucilage concentration from 2% to 6% *w*/*v*. This indicates that the mucilage can be used as a viscosity-imparting agent in suspension formulations.

**Figure 5:**
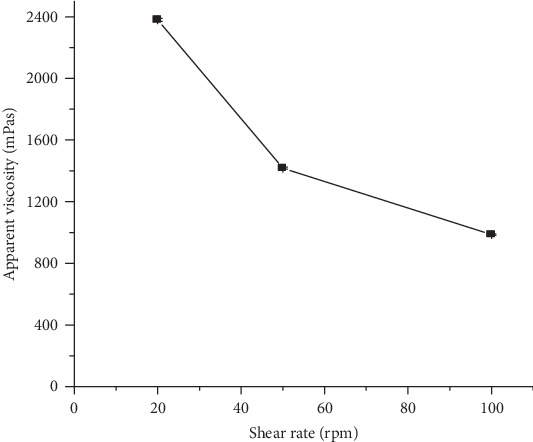
Apparent viscosity of *Grewia ferruginea* mucilage dispersions (4% *w*/*v*) at different shear rates.

*(2) Effect of Shear Rate on Viscosity*. As depicted in Figure 5, the viscosity of the mucilage decreased from 2380 ± 10 to 986.00 ± 5.29 mPas as the applied shear rate increased from 20 to 100 rpm. This demonstrates that the mucilage exhibited a pseudoplastic flow property. As the shear rate increases, molecules in a polymer chain get aligned in the direction of the flow resulting in a less interaction between the adjacent polymer chains. This property of pseudoplasticity is an important parameter to get uniform dosing for suspension formulations during administration [39].

#### 3.2.7. Phytochemical Analysis

Qualitative confirmation tests for secondary metabolites were carried out on the isolated mucilage of the *Grewia ferruginea* bark. The results of these tests revealed that steroids and tannins were present in the mucilage whereas saponins, flavonoids, and alkaloids were absent (Table 4).

#### 3.2.8. Evaluation of Acute Oral Toxicity

The acute toxicity studies of the mucilage showed no sign of toxic manifestations such as restlessness, respiratory distress, diarrhea, convulsions, and coma. No mortality was observed, and the behavioral pattern of the animals was unaffected during the observation period. Therefore, the mucilage was found to be safe up to 2000 mg/kg.

#### 3.2.9. Microbial Load Test

In BP [23], the recommended maximum tolerable limit of microbial load for pharmaceutical excipients is 1000 and 100 colony-forming units (cfu) per gram of sample for TVAC and TCMYC, respectively. In this study, evaluation of the extracted *Grewia ferruginea* mucilage for microbial load showed less than 87 cfu per petri dish for bacteria and 6 cfu per petri dish for fungi. All the controls used were free of any colony units. Therefore, the number of colony-forming units (cfu) per one gram of the sample was 870 cfu/g and 60 cfu/g for bacteria and fungi, respectively. This confirms that the microbial load of the mucilage was within the limit.

### 3.3. FTIR Spectroscopy of the Mucilage

**Figure 6:**
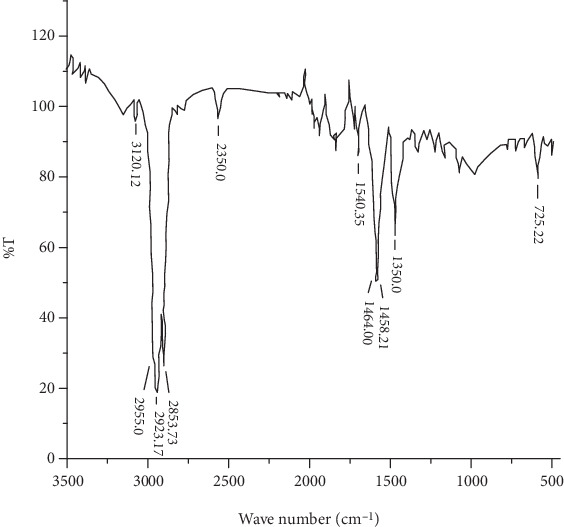
FTIR spectral characterization of GFM mucilage.

The infrared (IR) spectrum of a given compound is always unique and characteristic. As depicted in Figure 6, the mucilage showed sharp and characteristic peaks at 3120.12, 2955.0, 2923.17, 2853.37, 2350.0, 1540.35, 1464.0, 1458.21, 1350.0, and 725.22 cm^−1^. This indicates that the major polymeric functional and structural groups usually found in polysaccharides such as hydroxyl groups (3500-3100 cm^−1^), alkanes (3000-2850 cm^−1^), primary amine groups (1650-1580 cm^−1^), and carboxylic groups (1500-1800 cm^−1^) are also present in the mucilage.

### 3.4. Total Polysaccharide Content of the Mucilage

The calibration curve for the standard solution is presented in Figure 2, having a linear regression equation of *y* = 0.0003*x* + 0.0187 (where *y* is the peak area and *x* is the concentration in *μ*g/ml) with a correlation coefficient (*R*^2^) of 0.9963. As mucilage is a polysaccharide complex formed from sugar and uronic acid units, it can undergo hydrolysis into different monosaccharide units such as arabinose, galactose, glucose, mannose, xylose, and uronic acids upon the addition of strong acid. Accordingly, the total polysaccharide content of the mucilage was determined and found to be 60.6% (*w*/*v*). This is consistent with previous studies conducted on other mucilages [40].

## 4. Conclusion

Mucilage from the *Grewia ferruginea* bark was extracted, and various physicochemical properties of the mucilage were characterized based on specifications. Results showed that the mucilage possesses pseudoplastic rheological behavior, good powder flow property, and swelling power. Oral acute toxicity in mice showed that it is safe up to 2 g/kg body weight. Microbial load of the mucilage was also within the specified limit. These characteristics of the mucilage reveals its potential for use as an excipient in formulating modified release dosage forms as well as semisolid and liquid dosage forms like suspensions.

## Figures and Tables

**Table 1:** Solubility and swelling power of GFM at different temperatures (mean ± SD, *n* = 3).

Temperature (°C)	Swelling power (*g*/*g*)	Solubility (%)
25	64.03 ± 0.01	3.2 ± 0.2
40	64.5 ± 0.03	4.8 ± 0.1
55	72.66 ± 0.1	10.4 ± 0.2
65	73.05 ± 0.1	12.0 ± 0.3
75	73.2 ± 0.3	12.8 ± 0.1

**Table 2:** Solubility profile of *Grewia ferruginea* mucilage in different solvents.

Solvent	Solubility (g/ml)
Ethanol	0.0043 ± 0.01
Acetone	0.0004 ± 0.01
Chloroform	0.0007 ± 0.02
Cold water	Swellable
Hot water	Swellable

**Table 3:** Aqueous dispersion properties (conductivity and pH) of *Grewia ferruginea* mucilage at different concentrations (mean ± SD, *n* = 3).

Concentration (%/*w*/*v*)	pH (mean ± SD)	Conductivity (mean ± SD)
1	6.58 ± 0.01	1.94 ± 0.01
4	6.23 ± 0.01	10.32 ± 0.01
8	6.11 ± 0.01	16.64 ± 0.01
12	5.99 ± 0.01	22.20 ± 0.1

**Table 4:** Phytochemical confirmation tests on the isolated mucilage of the *Grewia ferruginea* bark.

Phytochemicals	Test	Results
Alkaloids	Wagner's test and Mayer's test	--
Steroids	Salkowski's test	++
Flavonoids	Alkaline reagent test (reaction with NaOH)	--
Tannins	Ferric chloride test	++
Saponins	Foam test	--

-- represents the absence and ++ represents the presence of the phytochemicals.

## Data Availability

The data used to support the findings of this study are included within the article.
